# Management challenges of supplementary feeding programs for severe acute malnutrition among children under five years: a qualitative study in Ethiopia

**DOI:** 10.1007/s44155-025-00256-8

**Published:** 2025-10-16

**Authors:** Ketema Degefa, Luisa Schneider, Freek Colombijn, Kedir Teji Roba

**Affiliations:** 1 Vrije Universiteit Amsterdam, Amsterdam, the Netherlands; 2 College of Health and Medical Sciences, Haramaya University, Harar, Ethiopia

**Keywords:** Severe acute malnutrition, Supplementary feeding programs, Management challenges, Children under five years, Ethiopia, Qualitative research

## Abstract

**Purpose:**

The study examines the challenges of implementing nutritional programs to address severe acute malnutrition in children under five, a major cause of illness and death in Ethiopia. This paper examines why nutritional programs are ineffective in Eastern Ethiopia.

**Methods:**

The research, conducted from April to June 2023 and from February to March 2024, included in-depth interviews, focus groups, and participant observations with mothers, healthcare workers, and traditional birth attendants. Thematic analysis was employed to identify key themes within the transcribed and coded data.

**Results:**

Five themes representing challenges in implementing nutritional feeding programs to address severe acute malnutrition among children under five were identified in the interviews, focus group discussions and observations. These themes are: management steps for severe acute malnutrition; the role of grandmothers in severe acute malnutrition treatment; the 1000-day approach to tackle malnutrition at early stages; behavioural and knowledge-building focused intervention; and factors influencing the management of SAM among health workers. The feeding program is hindered by too high workloads of the healthcare workers, limited access to healthcare facilities, inconsistent availability of nutritional supplements, and inadequate support for malnutrition. Delayed health-seeking behaviours pose behavioural challenges.

**Conclusions:**

The feeding program encounters challenges due to limited access to healthcare, inconsistent availability of nutritional supplements, inadequate support for malnutrition, and delayed health-seeking behaviour. Addressing malnutrition begins before a child’s birth and requires a comprehensive and long-term strategy. Intergenerational caregiving and effective collaboration between communities and healthcare workers can offer valuable insights.

## Introduction

The World Health Organization (WHO) defines severe acute malnutrition (SAM) in children aged 6 to 59 months as a low weight-for-height/weight-for-length ratio, bilateral pitting oedema, or low mid-upper arm circumference [1].Undernutrition refers to insufficient energy and nutrients required for maintaining good health [2]. Undernutrition consists of wasting (low weight-for-height), stunting (low height-for-age) and underweight (low weight-for-age) [2]. SAM affects 19 million children under five globally, leading to around 400,000 child deaths annually [3]. Also if child malnutrition does not lead to death, it is a severe issue, limiting their growth and cognitive development [4]. Africa has the highest number of SAM cases [5]. Malnutrition causes most child child deaths in sub Saharan Africa [6].

Ethiopia is one of the sub-Saharan countries where SAM is a major challenge [7]. According to the Ethiopian Mini Demographic Health Survey 2019, 37% of children under the age of five are stunted, with 12% severely stunted. Additionally, 7% of children are wasted, and 1% are severely wasted. Furthermore, 21% of children are underweight, including 6% who are classified as severely underweight [8]. The neonatal, infant, and under-five mortality rates in the five years preceding the 2019 survey were 33, 47, and 59 deaths per 1,000 live births, respectively [8]. Approximately 45% of deaths among children under five years old are directly or indirectly linked to undernutrition [8]. SAM was the third leading cause of mortality among children in Ethiopia, accounting for 8.1% of deaths [9, 10].

In Eastern Ethiopia, postmortem minimally invasive tissue sampling examination findings showed that SAM is the leading cause of death among children under five [11]. Several factors significantly influence the nutritional status of children in Ethiopia. Among children aged 6–59 months, large family size, age, diarrheal illness, and household food insecurity are notably associated with severe acute malnutrition [12]. Factors such as the child’s weight at birth, and the region of residence were significant determinants of malnutrition in children under five years old in Ethiopia [13]. Child malnutrition is influenced by factors such as insufficient food availability, and lack of sanitation and hygiene [14]. Despite the implementation of nutrition programs aimed at reducing malnutrition, the country is still falling short of global nutritional goals [15].

SAM can be prevented with early intervention and the Ethiopian government has set up several programs to that end. Already in 2003, Ethiopia launched its Community Health Extension Program, which comprised of 17 packages, including primary care services and child malnutrition management [16]. Health extension workers are responsible for community-based health promotion, including SAM management [17]. Unfortunately, as research has shown, these programs face challenges such as inadequate provision of ready-to-use therapeutic food, lack of antibiotics, and inappropriate exit from the program [18]. One of the challenges of SAM programs that is yet insufficiently understood is the implementation in the field in Ethiopia. For example, there is a misalignment between the biomedical and mothers’ understandings of SAM management challenges. Medical public health experts usually focus on the clinical management of SAM rather than social challenges. In contrast, mothers who attend health facilities for SAM management are more concerned about the social problems posed during the treatments. In contrast to the biomedical view used in most articles on malnutrition programs, this article does not examine limited resource access at the national level but focuses on the challenges of local healthcare workers to implement malnutrition programs, including the interactions with mothers and other community members. What kind of interactions take place on the ground between health care workers, mothers and other kin?Despite the potential of feeding programs which look good in their design, SAM continues to be a leading cause of child mortality. Notably, the management of SAM remains challenging due to inadequate healthcare, social barriers, and food shortages hindering the screening. Addressing the challenges in managing these programs is essential to render the feeding programs more efficient. This paper examines the challenges in implementing feeding programs, specifically at the level of health posts (the lowest healthcare facilities, in the hierarchy below health centres, nearby communities). Our study aims to shed light on effective SAM management in Eastern Ethiopia, utilizing insights from mothers and health workers and bridging the existing research gap.

## Methods

### Setting

We conducted the study at the Kersa Health and Demographic Surveillance System (HDSS) site in the Oromia region of Eastern Ethiopia. The Kersa district comprises 38 sub-districts, with 24 of them falling under the Kersa HDSS. The total population of the district is estimated to be 172,626 of whom 6.9% are urban dwellers people [19]. There are no data on SAM available at this level, but in a post-mortem child mortality surveillance it was found that severe malnutrition was the leading underlying cause of death in 15 out of 20 (75%) in children aged 28 days to 59 months [11].

### Design

We used a qualitative ethnographic design to explore the challenges of implementing nutritional programs. We employed a multi-method approach, collecting data through focus group discussions (FGD) and in-depth interviews (IDI). The first author also conducted separate participant observations (OBS) at community health centres and posts and in the homes of families who lost a child due to severe acute malnutrition. The use of multiple data collection tools facilitated data triangulation and validation.

Separate interview guides were developed for the focus groups and in-depth interviews. Open-ended questions were designed to allow participants to express their thoughts in their own words and bring up the issues that mattered most to them. The guiding questions were based on the specific research objectives. The guides incorporated and explained an information sheet about the purpose of the study before the interviews began. In addition to these guides, follow-up questions were posed to clarify specific aspects of the respondents’ answers. The research findings were presented using the Standards for Reporting Qualitative Research framework to ensure clear presentation [20].

### Participants and data collection

The study was conducted from April to June 2023 and February to March 2024 by the first author. Before data collection, the researcher underwent training on developing research questions, study design, qualitative data collection tools, participant selection, interview guide writing, data management, and data analysis approaches. The training helped the researcher to adopt a reflective approach and minimize subjectivity during data collection, coding, and analysis.

We used purposive sampling techniques to select participants to understand their diverse personal experiences. We selected mothers with children diagnosed with SAM, health workers working in health centres at nutritional stabilization centres, health extension workers working at community health posts, and traditional birth attendants.

A total of 44 interviews were conducted with the study participants. Interviews were conducted with mothers (n = 24), health workers (n = 8), grandmothers (n = 8) and traditional birth attendants (n = 4). Four focus group discussions were held with mothers and another four with health extension workers. Data collection was conducted in two rounds with different participants. In-depth interviews and focus group discussions took place in both rounds. We selected participants for the interviews and focus group discussions based on specific criteria. Our focus included pregnant women, lactating mothers, and mothers with children under five years old who are enrolled in nutritional feeding programs. Mothers of children under six months were also included. Additionally, health workers involved in these feeding programs were also included in the interviews and focus group discussions. The number of participants in the focus group discussion ranges between 7 and 10 participants, and the focus group discussions lasted 45–60 min.

The consent of interviewees and participants in the focus groups was obtained after we explained the study’s objectives. The guiding questions were prepared in English and translated into Afan Oromo (local language) to ensure all participants could understand and fully engage in the process. Afaan Oromo is the mother tongue of the interviewer and transcrips and the analysis were made in this language in order to stay as close to the views of the interlocutors; translations of quotes to English for this article were double checked.

The first author conducted participant observation at health centers and nutritional stabilization centers to understand the living circumstances of mothers with children suffering from SAM and their interactions with health workers during their stay. Additionally, we observed the distribution of food supplements for pregnant mothers, lactating mothers, and mothers with children under five who have SAM. The first author also conducted participant observations at the homes of these mothers to gain insight into their living situations, including cooking demonstrations led by health extension workers. We assured the participants that their information would be handled with care and kept confidential. No identifying information about them will be disclosed; all names mentioned in this paper are pseudonyms.

### Data analysis and interpretation

We analyzed and addressed the nutritional feeding management challenges within a health program using an iterative approach to data collection and ongoing analysis. All data collected through in-depth interviews and focus group discussions were digitally recorded and later transcribed. We transcribed the audio files verbatim in the original language (Afan Oromo). Based on the research objectives, data saturation was reached. To protect the participants’ privacy, we assigned them four-digit numbers and labeled the data types (Audio recordings and transcripts) using their initials instead of their names. The authors secured audio recorders in locked cabinets, and databases and computers were password-protected.

After conducting quality checks, we imported transcripts into NVivo, version 14, a software designed to manage and code large qualitative data sets. We used thematic analysis as our analytical approach to understand the views of study participants regarding the challenges of SAM management and their experiences at health facilities. Using an inductive approach, we coded the data from the transcripts. Initially, we read the transcripts several times to become familiar with the content. In the next step, we condensed the units of analysis, grouping and coding the data. Codes containing similar information were organized into categories, which were then further grouped into themes. Once we reached a consensus on these themes, we proceeded with the analysis. All the themes discussed in this paper are derived from the collected data.

### Strategies to enhance trustworthiness

We employed five steps to ensure the trustworthiness of the data: listening to the audio recordings multiple times, regularly discussing the coding framework and the emerging themes, using the same interview guide for all participants, capturing non-verbal communication, and thoroughly reviewing the objectives, methods, and procedures during the whole process. To enhance rapport, participants were given the option to choose a convenient location for their interviews and could withdraw if they felt uncomfortable. This approach helped build trust and encouraged participants to share their experiences openly. Transferability was achieved through purposive sampling, which allowed for a diverse range of perspectives from a wider collection of participants. This strategy facilitated a comprehensive understanding of the participants’ viewpoints.

## Results

### Themes emerged during data analysis

Five themes emerged from the study: Steps for managing severe acute malnutrition; the role of grandmothers in severe acute malnutrition treatment; 1000 days approach to tackle malnutrition at early stages; behavioural and knowledge-building focused intervention and factors influencing the management of SAM among health workers. The table below explains the themes that emerged during the data analysis, including participants’ quotes selected because they concisely capture the theme (Table 1).

**Table 1:** Sample of quotes organized by theme

Theme	Quotes
Steps for managing severe acute malnutrition	‘We conduct monthly outreach screenings alongside other healthcare services, mobilizing mothers in a neighbourhood to gather pregnant and lactating women and under-five children to ensure comprehensive screenings’ (a health care worker at a health post) ‘Screening and Outpatient Therapeutic Programs (OTPs) are typically for children aged between six and 59 months. Infants under six months are not always brought in for SAM screening. If an infant under six months is identified as undernourished, the treatment is provided to the mother, rather than the child’ (a health care worker at a health post) ‘Children with poor appetite, medical complications, severe wasting, and bilateral pitting edema are referred to a health center for further treatment. Infants 0–6 months with medical complications, recent weight loss, failure to gain weight, ineffective feeding, or any medical or social issue needing detailed assessment or support are also referred. SAM cases with good appetite, no medical complications, bilateral pitting edema, and severe wasting are eligible for OTP at the health post.’ (a health care worker at a health center) "’SAM cases with good appetite, no medical complications, bilateral pitting edema, and severe wasting get Plumpy’nut and their mothers nutritional counseling until they reach their target weight.’ (a health care worker at a health post) ‘The living conditions and transportation to the stabilization centre for SAM treatment can be challenging; I often delay finding transportation and arranging food for my children at home, which they eat while I am away for my child treatment.’ (a mother at a health center)
The role of grandmothers in severe acute malnutrition treatment	‘My child’s wife recently gave birth, and she is unable to bring her older child for this treatment [at the stabilization centre]. I want to support them because he is also my grandchild, but I am not familiar with healthcare or the different types of formula milk for a child.’ (a grandmother at a health center) ‘They [the grandmothers] provide butter to infant children as a form of medication, believing it can help prevent stomach pain caused by parasites.’ (a health care worker at a health post) ‘Commonly, grandmothers provide guidance on pregnancy-related concerns, including labour and pregnancy-related sickness, to newly married mothers, drawing from their lived experiences.’ (a traditional birth attendants at a village)
1000 days approach to tackle malnutrition at early stages	‘If a malnourished young woman does not receive proper nutrition during her adolescent years and later becomes pregnant, her child has a higher risk of being underweight and malnourished.’ (a health care worker at a health center) ‘Childcare is a social role given to mothers, while men generate income.’ (a community member at village)
Factors influencing the management of SAM among health workers	Tadu said: ‘I worked as a health extension worker for 15 years in the same position. I got married and have children. I feel tired and think it would be better to find a new position in an office rather than continue as a health extension worker. It would be good to bring new staff with fresh energy to take on this role.’ (a health care worker at a health post) ‘Someone who has been working in the village for many years might be viewed as a member of the community and people may no longer take their advice seriously’ (a health care worker at a health post) We are here today to receive supplementary food support for our severely malnourished children, but the health workers informed us that it is out of stock.’ (a mother at a health post)
Behavioral and knowledge-building focused intervention	‘Health extension workers are responsible for food demonstration events, health education, family planning, vaccine provision, and treatment tasks. We often travel on foot door to door to sensitize mothers to vaccination and nutritional screening, which is difficult to do regularly.’ (a health care worker at a health post)

### Steps for managing severe acute malnutrition

Healthcare workers emphasized that nutritional screening is essential for mothers and children, particularly pregnant and lactating women and children under five. Mothers who visit health posts for immunization or family planning services undergo nutritional screening. They are advised to maintain a healthy lifestyle if the results are good. However, if the screening indicates malnutrition, they may be referred to a feeding program or directed to seek further treatment at health posts or centres.

Health workers adhere to WHO guidelines to assess the nutritional status of children and screen for swelling and mid-upper arm circumference (MUAC). They weigh children using a scale to calculate age-specific metrics and enrol eligible children in the Outpatient Therapeutic Programme. Additionally, health workers conduct monthly outreach screenings and provide other healthcare services. They mobilize mothers in the community to gather pregnant and lactating women, as well as children under five years old, ensuring comprehensive screenings for all. Nutritional screening is a routine activity for health workers at health posts; however, local people are unsatisfied with screening without significant interventions.

Chaltu works at a Stabilization Centre that offers inpatient admission for children with acute cases of SAM. Children are admitted with their mothers and receive formula milk until they have recuperated. She described the findings of the community screening in the following manner: ‘Malnutrition is prevalent among children under the age of five. Mothers often have no option but to leave their infants unattended at the sabilization centre while carrying out errands, leading to inadequate child care. (IDI: Diploma in Nursing; 23 years old woman).

Chaltu continued: ‘Screening and Outpatient Therapeutic Programs are typically for children aged between six and 59 months. Infants under six months are not always brought in for SAM screening. If an infant under six months is identified as undernourished, the treatment is provided to the mother rather than the child’. The healthcare worker, Tigist, explained that screening is not conducted every month as planned. It is sometimes cancelled when they [the health workers] receive calls for meetings, attend training sessions, or are occupied with emergency tasks, such as managing an ongoing cholera outbreak’. (OBS: Health worker; 25 years old woman). This irregular screening is a pressing issue and highlights the urgent need for improved and consistent screening practices.

Health workers strongly agree on the relationship between screening for nutritional status and breastfeeding. Fatuma, for instance, elucidated that: 'Many children under two are screened for SAM due to inadequate breastfeeding, which does not only hamper their development but also increases their vulnerability to bacterial infections’. (FGD: Health worker; 28 years old woman). Tigist also stressed the significance of breastfeeding and caring for children under two years old: 'Breastfeeding for up to six months, followed by the introduction of supplementary feeding with proper hygiene and care is crucial for child health and survival. If children do not receive regular breastfeeding during the first six months, they are at high risk of experiencing SAM’. The lack of clean water in villages also threatens children’s nutritional status. A mother pointed out that children drink unclean spring water in some villages. (IDI: traditional birth attendant; 55 years old woman). Other mothers explained that the lack of clean water, poor sanitation, and hygiene may contribute to SAM in children. (OBS: mothers at health posts).

After screening, health extension workers categorize malnutrition cases on a case-by-case basis. Based on our interviews with health extension workers, children aged 6–59 months with bilateral pitting or severe wasting (MUAC < 11.5 cm) are diagnosed with SAM and require medical attention. MAM, or moderate wasting, is identified in children 6–59 months old with a MUAC measurement of ≥ 11.5 cm and < 12.5 cm’. Another health workers. Lense, added: 'MUAC is used for identifying SAM (red), MAM (yellow), and good nutritional status (green). MAM children are eligible for outpatient treatment, while SAM children with additional complications require inpatient care at a health centre.’ Most community health workers highlighted that most children admitted to health posts have red, some yellow, and a few green MUAC tests. This variation shows that decisions are not merely based on the MUAC test and require expert judgement of the health workers.

Jemal, a health worker at a stabilization centre, is explaining the treatment process. He said: 'If a child’s MUAC falls within the yellow range, mothers receive only nutritional advice’. (IDI: Diploma in Nursing; 29 years old married man). However, if a child’s MUAC falls within the red range, the child receives treatment for SAM. The next step involves assessing the child’s appetite using Plumpy’nut, a well-known Ready-to-Use-Therapeutic-Food of peanut paste in a plastic wrapper, and observing their eating habits. If the child fails the appetite test, a referral letter is sent for further inpatient treatment at the Stabilization Centre’. (OBS: During mothers’ visits health posts).

Most mothers feel stressed when hearing the screening result, but they ultimately accept it. They tend to blame themselves when their children are diagnosed with SAM. However, many mothers show a proactive approach, eagerly seeking support from health posts or centres when their children are screened. Others, who have received support, share the nutritious supplementary food provided by the health posts with other healthy children at home. Health worker Firani said: ‘We usually advise mothers to use therapeutic food for malnourished children, but we often hear that they also share it to healthy children. (FGD: Health worker; 29 years old woman).

Health extension workers highlighted that they select the appropriate treatment following the classification of children with severe acute malnutrition into two groups based on severity. Kedija explained the treatment process as follows: ‘Children with poor appetite, medical complications, severe wasting, and bilateral pitting edema are referred to a health center for further treatment. Infants 0–6 months with medical complications, recent weight loss, failure to gain weight, ineffective feeding, or any medical or social issue needing detailed assessment or support are also referred. (IDI: Health worker; 35 years old woman). When a mother with a child under six years old is malnourished, she is referred to a health center from the health post. Halima explained: ‘I gave birth by c-section because of medical complications. We don’t have enough resources to eat and breastfeed. During a community visit, health workers screened me and told me I’m malnourished.’ (FGD; a 19-year-old woman with a two-month-old child). The severity of malnutrition determines the type of care and location of treatment.

The latter category consists of children receiving treatment during weekly visits to the health post. Fatuma told us, ‘SAM cases with good appetite, no medical complications, bilateral pitting edema, and severe wasting get Plumpy’nut and their mothers nutritional counseling until they reach their target weight.’ (IDI: Health worker; 38 years old woman). For complicated cases, they receive treatment at the health center, including formula milk and medications. After recovering, children are sent back to the village health post for further treatment until they recover entirely from SAM. However, sometimes, children re-join the program before fully recovering and show malnutrition symptoms again.

Health worker Misira said: 'The severity of a child’s malnutrition can change within a few days, often unpredictably, based on the quality of care they receive and the availability of resources. This means that a child could quickly progress from SAM with complications to SAM without complications or vice versa. Mothers’ delay in seeking healthcare for SAM treatment also changes the types of SAM.' (IDI: Health worker; 35 years old woman).The analytical distinction between categorization and treatment can in practice not be strictly made.

Health workers highlighted that, unfortunately, the first symptoms of SAM are not always apparent to mothers. Data during observation at health posts shows that some mothers delay seeking treatment for malnutrition because they think that some of the symptoms are just part of the usual challenges of children growing up. For example, Bontu has children with SAM and did not recognize the early symptoms. Her daughter stayed at home with SAM for a month because she thought those symptoms were usual for every child during their childhood. She visited the health centre when the SAM cases had progressed, and her daughter had many wounds on different parts of her legs, which required intensive care, beyond the capacity of health centers. (OBS: During mothers’ visits health posts). Many mothers have similar beliefs, which lead to low health-seeking behaviour.

Management of SAM cases with medical complications, such as Bontu’s child, involves treatment at a Stabilization Centre. A stabilization Centre is a part of a health center but there is a separate room for SAM treatment. For a child to be eligible for inpatient admission, the health extension workers must write a referral letter to the focal person at the Stabilization Centre. Some mothers who were referred to the health centre visit the Stabilization Centre at the health center, but not all do. A mother named Alami expressed that: ‘the living conditions and transportation to the stabilization centre for SAM treatment can be challenging; I often delay [first] finding transportation and arranging food for my children at home, which they eat while I am away for my child treatment.’(IDI: A 32-year-old woman who is a mother with children aged 4 years).

While the various steps to take measures for children with varying degrees of SAM are clear on paper, the actual process can be different. Some of the mothers take the referral letter and for various reasons return home without treatment of their children. In informal conversations with them, I learned that some have many children at home who need care, some lack transportation and money, and some are pregnant or sick and therefore cannot travel. (OBS: During mothers’ visits health center). In these circumstances, they may visit traditional healers near their village instead or delay accessing healthcare. Mothers often face the dilemma; in choosing to visite health centers with one or some of their children, they also choose to leave their other children at home. Factors such as distance from the health centers (mothers often travel two–three hours on foot) and their own health conditions influence whether or not they continue going. (OBS: During mothers’ visits health center).

It is important to understand that the majority of mothers let time pass before visiting a Stabilization Centre after receiving the referral letter. This delay not only worsens the severity of SAM but also significantly impacts the health and well-being of the child, potentially leading to death.

When a child with complicated SAM is admitted for inpatient services, they may stay for up to seven days, depending on the child’s immunity and the quality of care they receive from the Stabilization Centre. According to Jemal, already cited above, children with severe edema and complications receive F-75 formula milk for a few days. F-75 is considered a starter formula with 75 kcal energy and 0.9 gm protein per 100 ml. Typically, children recover from edema and complications within two days and during this time, they start catching up with F-100 formula, which provides 100 kcal and 2.9 g protein per 100 ml. The catch-up formula is used when children experience less severe complications and need time to recover. (OBS: During mothers’ visits health center).

Just like mothers face the dilemma to leave home to go to a health centre, they are considering whether they should stay once they are in. Depending on the severity, some children may require inpatient services for up to two weeks. Children with SAM and medical complications such as vomiting, coughing, and diarrhea take longer to recover. When mothers are referred to a health center, they are forced to adapt to a new living environment. (OBS: During mothers’ visits health center). One challenge mothers often face is having to leave their other children at home and staying at the health center with the child who is under treatment. As a result, mothers may choose to leave without officially informing the Stabilization Centre staff, slipping out quietly with their child.

### The role of grandmothers in severe acute malnutrition treatment

When mothers feel they cannot stay at the health center, because they have children at home, they may ask female kin to take care of their child at the health centre, and the most frequent support comes from grandmothers. (OBS: During mothers’ visits health center). Subsequenly, grandmothers do not only play a role in the feeding of the young children, but also in the management of malnutrition programs. It is common that mothers seek help when they are pregnant, sick, or have recently given birth. A grandmother said, ‘My child’s [son’s] wife recently gave birth, and she is unable to bring her older child for this treatment. I want to support them because he is also my grandchild, but I am not familiar with healthcare or the different types of formula milk for a child.’ (IDI: 48 years old women at health centre). Grandmothers from either the wife’s or husband’s side take on this responsibility and they can also stay with their grandchild with SAM at a health centre.

There are differences in feeding traditions between mothers and grandmothers that span across generations. Grandmothers tend to have a better knowledge of a variety of foods and advise mothers to consider feeding their children with foods that promote health and strength, such as those containing vitamins, proteins, and carbohydrates. (OBS: During mothers’ visits health center). Mothers, in contrast, are more likely to practice bottle feeding, often due to their work schedules. This can make it difficult for mothers to consistently provide a diverse range of foods to their children. Moreover, grandmothers have learned local practices for fattening and the transition to solid food which they apply to their grandchildren. For instance, grandmothers may recommend giving small portions of butter to infants, believing that it makes children stronger and prepares them for solid food later in life. (OBS: During mothers’ visits health center). Fatu, a health worker explained: ‘They provide butter to infant children as a form of medication, believing it can help prevent stomach pain caused by parasites.’ However, health workers do not recommend feeding butter to children before they are two years old. (IDI: health worker, 35 years old married woman).

Grandmothers sometimes have difficulties adapting to feeding programs and a certain flexibility of the health centers is crucial in this respect.The treatment process from screening to the feeding program is not straightforward, and the grandmothers often struggle, particularly with formula milk feeding. (OBS: During mothers’ visits health center). It would help if the procedure is simplified and the health centre provides comprehensive support to make it more understandable to them. The intergenerational knowledge gap can make it difficult for some grandmothers to comprehend the feeding program.

Grandmothers find the health center environment unappealing, which is another challenge for them. They are expected to stay at the health center for seven to fourteen days, depending on the child’s condition. During their stay, they must take care of their own feedings needs and either cook for themselves or have their families provide food. (OBS: During mothers’ visits health center). Previously, an NGO used to support them by offering spaghetti to grandmothers or mothers staying at the health center. However, now they are expected to buy groceries for themselves. The risk is that some grandmothers do not have enough money to buy food, and they may struggle to find time to prepare their meals. In this situation, they need equipment and food to prepare and they are dependent on someone else to bring cooked food or ingredients or utensils to cook, which adds to their insecurity. Sometimes they receive informal support from the health workers residing around the health center.

The intergenerational knowledge gap is not always a challenge. It can also be an opportunity. Ideally, grandmothers and mothers exchange their knowledge. Traditional birth attendants said that: ‘commonly, grandmothers provide guidance on pregnancy-related concerns, including labour and pregnancy-related sickness, to newly married mothers, drawing from their lived experiences.’(IDI: a mother 46 years old). Grandmothers also share knowledge on breastfeeding practices, including when to start and stop feeding. Finally, when a newly born baby is sick, grandmothers are usually the first to be called for advice before visiting a healthcare facility. In cases where the baby needs a massage or herbal remedy, grandmothers are often the ones to provide it.

### 1000 days approach to tackle malnutrition at early stages

So far, we have discussed interventions to address acute malnutrition, but the government also runs another program called the "1000 days approach" from the conception of a child until the child reaches the age of two.The 1000 day approach is premised on the early idenitificatoin of a pregnancy. A key component of this stretgy is the role of community volunteers as pregnancy detectors, who connect expectant mothers with health centers and promote healthy pregnant practices. The government is providing two types of solutions during these thousand days: provision of Plumpy’nut and soya bean flour and education/knowledge-building interventions. The strategy also includes education on food supplementation, regular check-ups, and antenatal care services. However, our research reveals two potential issues that could jeopardize the 1000 days intervention.

First, the 1000-days-approach primarily focuses on mothers as the intervention target. But often it is the men who take decisions about resource allocation, and this is a risk when resources might be limited, while women face increased nutritional requirements during pregnancy and breastfeeding. Women are the main receivers of health information and nutritional guidance, encouraging them to exclusive breastfeeding practices. Husbands prioritize economic responsibilities and are less involved in family health and less aware of extra nutritional needs of mothers during pregnancy and breastfeeding. Abel, for instance is convinced that: ‘childcare is a social role given to mothers, while men generate income.’ Men should not only support mothers during pregnancy and thereafter by generating income, but also by taking children to health centers for vaccinations when mothers are unable to. (IDI: 34 years old man).

To give an example, during a healthcare visit, 20-year-old mother Kedija discovered that her eight months old daughter was malnourished. Kedija had already lost two infants and suspected that her daughter’s malnutrition may be due to her own nutritional deficiencies during pregnancy and lactation. Economic problems made it difficult for her to access diverse foods during pregnancy and thereafter. Although Kedija’s daughter’s nutritional deficiency was improving at the time of research, gaining weight from 5.5 to 6.2 kg, Kedija was not reassured. She was unwell during her first two deliveries, and both infants died at early stages. (OBS: During mothers’ visits health center. This added to her frustration and worries for the current child, as her grief remains visible.

Second, The first 100 days of a child’s life are crucial for optimal health and growth. However, effectively addressing SAM challenges requires including children up to five years old in this approach.

To add to the various social issues of mothers, other kin and health workers, there are also practical logistics that haunt the management of the nutrition program. The intervention aims to provide Plumpy’nut and Corn-Soy Blend (CSB) flour as part of a feeding program for malnourished children and pregnant or breastfeeding mothers. Children with MAM were supposed to receive ready-to-use supplementary food, but due to supply issues, this is no longer available. Children with SAM without complications aged over six months are provided with ready-to-use therapeutic food, with supply for a week until the child recovers. The amount of sachets provided depends on the nutritional status of the child, with each sachet containing 500 kcal. Every week, mothers bring their children to the health post for the feeding program, where health extension workers check and update the nutritional chart to monitor the child’s progress. As the child’s nutritional status improves, the number of sachets received decreases. (OBS: During mothers’ visits health posts).

However, Plumpy’nut is often out of stock, leading to complications in the child’s condition. A mother with a severely malnourished child without complications at health posts said: ‘We are here today to receive supplementary food support for our severely malnourished children, but the health workers informed us that it is out of stock.’ CSB flour should be given to pregnant and breastfeeding mothers, but, as health workers explained, it is often given only once or twice a year due to supply shortages. Pregnant and lactating mothers and children under five are routinely screened for malnutrition, but almost half of them do not receive support due to demand outweighing supply. Providing resources for children and mothers remains a challenge due to the high number of monthly SAM cases and irregular treatment aid provided by non-governmental organizations. In short, the demand for support often outweighs the supply, leading to delays in receiving assistance, especially for breastfeeding and pregnant mothers. (OBS: During mothers’ visits health center).

### Behavioral and knowledge-building focused intervention

The health extension workers are also responsible for organizing pregnancy conferences. These sessions focus on nutrition, breastfeeding, and health education. For instance, during these pregnancy conferences, cooking demonstrations are held to raise knowledge of food groups, vitamins, minerals, and nutrition for mothers and children. Health extension workers organize these demonstrations at health posts: they provide items like vegetables, fruits, and eggs, and teach mothers how to prepare diverse meals for children. The cooking demonstrations significantly increase awareness about a balanced diet, but many mothers struggle to provide this variety regularly at home.

Unfortunately, these important meetings are often irregular due to human capital shortages and financial constraints in health posts. Often, only two extension workers are responsible for these activities, along with family planning, vaccine provision, and treatment tasks. Health worker Kume said ‘health extension workers are responsible for food demonstration events, health education, family planning, vaccine provision, and treatment tasks. We often travel on foot door to door to sensitize mothers to vaccination and nutritional screening, which is difficult to do regularly’. (IDI: a 27 year old health worker). The feasibility of providing and sustaining health education is limited due to these resources. Health extension workers also face additional challenges, such as traveling without transportation (on foot), and the implementation of these interventions requires time and effort. These demonstrations should occur every three-four months but are irregular due to the busy schedules of the health extension workers. Moreover, also the mothers often do not find the time to attend them.

### Factors influencing the management of SAM among health workers

The working conditions of health workers and the limited supplies are significant factors contributing to the challenges for SAM management. We should not only take the lives of mothers, grandmothers and fathers into account, but also the social conditions of health workers. There are two to three health extension workers assigned at the health post who constantly reside in villages, speak the local language, complete secondary school, and serve the communities. Health extension workers provide health education, basic services, and awareness about hygiene, family planning, antenatal care, nutritional counselling, breastfeeding, and facility delivery. They treat SAM without complications and implement health services and community outreach activities. They collaborate with local volunteer mothers, providing curative, promotional, and preventative health services through home visits and outreach activities.

The workload weighs heavily on the health workers for various reasons, but with the same outcome that they do not have enough time for children with SAM. While initially, three female health workers were assigned to each village, turnover and resource constraints led to several villages having only one or two health worker. Health workers face heavy workloads, lack of career prospects, poor working conditions, insufficient salaries and housing, and high inflation rates, which affect their daily lives and their families’ needs.

Many health extension workers who have been in the same position for over ten years are dissatisfied with their employment and wish to pursue other opportunities. Tadu said: ‘I worked as a health extension worker for 17 years in the same position. I got married and have children. I feel tired and think it would be better to find a new position in an office rather than continue as a community worker. (FGD: a 38 years old health worker). It would be good to bring new staff with fresh energy to take on this role.’ In the same vein, Iftu, who has been a health extension worker for 15 years, recommends to seek new staff members for these responsibilities. (FGD: a 36 years old health worker). She pointed out that: ‘Someone who has been working in the village for many years might be viewed as a member of the community and people may no longer take their advice seriously’. Replacing health workers too frequently could hinder malnutrition treatment, as it requires more focus on family education and building a following. Their numerous job responsibilities prevent them from dedicating more time to nutrition management.

The implementation of nutritional programs is significantly impacted by health worker burnout. Fantu health workers said, ‘We are burned out working in the village for many years, and young workers should take on these responsibilities.’ (FGD, health worker married woman). Health extension workers are experiencing burnout due to limited resources and high caseloads. They feel powerless, blame themselves for shortcomings, and struggle to deliver necessary activities. The devastating impact of SAM, particularly on children with deteriorating conditions, exacerbates work pressure and emotional burdens, further burdening them.

## Discussion

SAM remains a significant public health challenge in Ethiopia, despite an integrated policy for screening, registration, intervention, and treatment. In Ethiopia, approximately 45% of under-five child mortality cases are linked to undernutrition, with SAM accounting for 8% of these deaths [12]. The proportion of deaths associated with malnutrition in this district was notably high [21]. Though the different feeding programs look good on paper, they do not always run smoothly. In this article we have aimed at unveiling management problems at health posts and health centers, that is, at the level where the program meets the targeted beneficiaries. What are the challenges at this important link in the development chain? The efficiency and effectiveness in tackling SAM remain challenges because of several reasons.

At a national scale, the program’s effectiveness is hindered by limited supplementary food supplies, such as Plumpy’nut for children and soya bean flour for pregnant and breastfeeding mothers. Often, the feeding program’s supply is provided by international non-governmental organizations. When the feeding supply is available, the nutritional condition of children and mothers is better.

The second problem is the workload of health extension workers affecting the nutritional program implementation and SAM management. The high workload, sometimes even resulting in burnout of the health workers, which has been observed in other parts of Ethiopia as well [22, 23] and our study both confirms and deepens this insight. Service distribution limitations, unsatisfactory career structures, poor working conditions, inadequate salary and budget, and excessive working hours all lead to fatigue, frustration and, sometimes, burnout. As a result of the unappealing work structures, health extension workers intention to quit the job is high. Our study has revealed that health extension workers were often required to perform tasks from other sectors, leaving them with less time to complete their own regular activities. We demonstrated that health extension workers struggle to meet the demands of the feeding program, leading to feelings of ineffective performance, self-doubt, burnout, and job loss. The lack of support they receive from higher officials also limits their performance capacity [24] and understanding of stakeholder engagement practices in multisectoral nutrition, which is vital for effective program management [25].

Grandmothers play a crucial role in community maternal and child nutrition programs and are seen as agents of change in Western Africa [26], but their contribution has so far been ignored in studies on Ethiopia. Grandmothers play a crucial role in treating malnourished children, but often struggle with formula milk feeding. Simplifying procedures and providing comprehensive support can help grandmothers understand the program. Grandmothers play a critical role in decision-making, contributing to the survival of children in managing malnutrition. Engaging family members in these nutritional initiatives may lead to better health-seeking behaviours for SAM patients and ensure the sustainability of the interventions. The intergenerational knowledge gaps between mothers and grandmothers hinder food diversity, but can also provide opportunities for sharing knowledge, guidance, and support. These findings, supported by another study, emphasize the importance of involving grandmothers in nutritional programs to promote recommended behaviours and improve program sustainability [27].

## Conclusion

The study highlights that the feeding program faces challenges due to limited healthcare access, inconsistent nutritional supplement availability, inadequate malnutrition support, and delayed health-seeking behaviour. We emphasize the need for adequate resources and a workforce to detect and treat cases promptly. SAM can be reduced by empowering health extension workers, improving work structures, utilizing local resources, balancing workloads, and developing community-centred approaches. A comprehensive intervention focusing on behaviour and knowledge-building is essential for preventing SAM in early childhood and reducing the additional treatment costs. It is important to note that malnutrition issues begin before a child is born, necessitating a holistic and longitudinal approach. Addressing system-related challenges and behavioral-related issues together would foster policy adjustments that successfully address the SAM.

### Limitations of the study

The study, conducted in Eastern Ethiopia, has findings that are significant for SAM in that region. However, the results may not be nationally applicable. Even in Eastern Ethiopia the study could be replicated at a wider scale if more research funding became available. A larger research could also include higher levels in the health centres hierarchy and their views on the malnutrition programs at the local level. Our study was based on a snapshot approach; a longitudinal study would show whether solutions are sustained and challenges overcome, or aggravated, over a longer time. This paper highlights the challenges of managing SAM despite the existing protocols and manuals. We demonstrated how irregularities in feeding programs and behavioural barriers impede SAM management. Additionally, the burnout of healthcare workers due to resource constraints and poor living conditions poses another challenge to addressing SAM. We recommend conducting a nationwide, multimodal study at all levels of the health system to understand better the challenges associated with SAM, which can inform policy development.

## Data Availability

The data supporting the findings of this study are available upon request from the corresponding author (KD). Email: k.degefa@vu.nl. The data are not publicly available due to containing sensitive information that could compromise the privacy of research participants.

## References

[1] World Health Organization. Identification of Severe Acute Malnutrition in Children 6–59 Months of Age. 2014.

[2] Maleta K. Undernutrition. Malawi Med J. 2006;18:189–205. 27529011 PMC3345626

[3] World Health Organization. Pocket book of hospital care for children: guidelines for the management of common childhood illnesses. World Health Organization; 2013. 24006557

[4] Suryawan A, Jalaludin MY, Poh BK, Sanusi R, Tan VMH, Geurts JM, et al. Malnutrition in early life and its neurodevelopmental and cognitive consequences: a scoping review. Nutr Res Rev. 2022;35:136–49. 34100353 10.1017/S0954422421000159

[5] World Health Organization. Joint child malnutrition estimates-levels and trends (2019 edition). WHO World Health Organization. 2018.

[6] Adeyeye SAO, Ashaolu TJ, Bolaji OT, Abegunde TA, Omoyajowo AO. Africa and the Nexus of poverty, malnutrition and diseases. Crit Rev Food Sci Nutr. 2023;63:641–56. 34259104 10.1080/10408398.2021.1952160

[7] Anato A. Severe acute malnutrition and associated factors among children under-five years: A community based-cross sectional study in Ethiopia. Heliyon. 2022;8. 10.1016/j.heliyon.2022.e10791 PMC9529577 36203897

[8] Ethiopian Public Health Institute (EPHI) [Ethiopia] and ICF. 2021. Ethiopia Mini Demographic and Health Survey 2019: Final Report. Rockville, Maryland, USA: EPHI and ICF.

[9] Kebede F. Severe Acute Malnutrition (SAM) associated mortality rate of children attending HIV/AIDS care in North West Ethiopia, 2009–2019. SAGE Open Medicine. 2022;10:20503121221081336. 35251656 10.1177/20503121221081337 PMC8891843

[10] Oumer A, Mesfin L, Tesfahun E, Ale A. predictors of death from complicated severe acute malnutrition in East Ethiopia: survival analysis. IJGM. 2021;14:8763–73. 10.2147/IJGM.S337348 PMC8627852 34853530

[11] Madrid L, Alemu A, Seale AC, Oundo J, Tesfaye T, Marami D, et al. Causes of stillbirth and death among children younger than 5 years in eastern Hararghe, Ethiopia: a population-based post-mortem study. Lancet Glob Health. 2023;11:e1032–40. 37271163 10.1016/S2214-109X(23)00211-5 PMC10282072

[12] Anato A. Severe acute malnutrition and associated factors among children under-five years: a community based-cross sectional study in Ethiopia. Heliyon. 2022;8: e10791. 36203897 10.1016/j.heliyon.2022.e10791 PMC9529577

[13] Yirga AA, Mwambi HG, Ayele DG, Melesse SF. Factors affecting child malnutrition in Ethiopia. Afr H Sci. 2019;19:1897. 10.4314/ahs.v19i2.13 PMC6794502 31656473

[14] Hutton G, Chase C. The knowledge base for achieving the sustainable development goal targets on water supply. Sanitation and Hygiene IJERPH. 2016;13:536. 27240389 10.3390/ijerph13060536 PMC4923993

[15] Amare ZY, Ahmed ME, Mehari AB. Determinants of nutritional status among children under age 5 in Ethiopia: further analysis of the 2016 Ethiopia demographic and health survey. Global Health. 2019;15:62. 31694661 10.1186/s12992-019-0505-7 PMC6836473

[16] Assefa Y, Gelaw YA, Hill PS, Taye BW, Van Damme W. Community health extension program of Ethiopia, 2003–2018: successes and challenges toward universal coverage for primary healthcare services. Global Health. 2019;15:24. 30914055 10.1186/s12992-019-0470-1 PMC6434624

[17] Mangham-Jefferies L, Mathewos B, Russell J, Bekele A. How do health extension workers in Ethiopia allocate their time? Hum Resour Health. 2014;12:61. 25315425 10.1186/1478-4491-12-61 PMC4209031

[18] Tadesse E, Ekström E-C, Berhane Y. Challenges in implementing the integrated community-based outpatient therapeutic program for severely malnourished children in rural southern Ethiopia. Nutrients. 2016;8:251. 27128936 10.3390/nu8050251 PMC4882664

[19] Assefa N, Oljira L, Baraki N, Demena M, Zelalem D, Ashenafi W, et al. HDSS profile: the kersa health and demographic surveillance system. Int J Epidemiol. 2016;45:94–101. 26510420 10.1093/ije/dyv284 PMC4795560

[20] O’Brien BC, Harris IB, Beckman TJ, Reed DA, Cook DA. Standards for reporting qualitative research: a synthesis of recommendations. Acad Med. 2014;89:1245–51. 24979285 10.1097/ACM.0000000000000388

[21] Madewell ZJ, Keita AM, Das PMG, Mehta A, Akelo V, Oluoch OB, et al. Contribution of malnutrition to infant and child deaths in Sub-Saharan Africa and South Asia. BMJ Glob Health. 2024;9:e017262. 10.1136/bmjgh-2024-017262 PMC11624724 39638608

[22] Kitila KM, Wodajo DA, Debela TF, Ereso BM. Turnover intention and its associated factors among health extension workers in illubabora zone, south west Ethiopia. J Multidisciplinary Healthcare. 2021;1609–21. 10.2147/JMDH.S306959 PMC8254187 34234449

[23] Zerfu TA, Tareke AA, Biadgilign S. Challenges and experience of the Ethiopian rural health extension program: implications for reform and revitalization. BMC Health Serv Res. 2023;23:1309. 38012613 10.1186/s12913-023-10253-9 PMC10683286

[24] Okwaraji YB, Hill Z, Defar A, Berhanu D, Wolassa D, Persson LÅ, et al. Implementation of the ‘optimising the health extension program’ intervention in Ethiopia: a process evaluation using mixed methods. IJERPH. 2020;17:5803. 32796574 10.3390/ijerph17165803 PMC7459764

[25] Warren AM, Constantinides SV, Blake CE, Frongillo EA. Advancing knowledge about stakeholder engagement in multisectoral nutrition research. Glob Food Sec. 2021;29: 100521.

[26] MacDonald CA, Aubel J, Aidam BA, Girard AW. Grandmothers as change agents: developing a culturally appropriate program to improve maternal and child nutrition in sierra leone. Curr Develop Nutrition. 2020;4:nzz141. 10.1093/cdn/nzz141 PMC6932963 31893262

[27] Lowery CM, Craig HC, Litvin K, Dickin KL, Stein M, Worku B, et al. Experiences engaging family members in maternal, child, and adolescent nutrition: a survey of global health professionals. Curr Develop Nutrition. 2022;6:nzac003. 10.1093/cdn/nzac003 PMC8866103 35224418

